# The Incidence Of Prescribing Errors In An Eye Hospital

**DOI:** 10.1186/1471-2415-5-4

**Published:** 2005-03-22

**Authors:** K Mandal, SG Fraser

**Affiliations:** 1 Sunderland Eye Infirmary, Queen Alexandra Road, Sunderland SR2 9HP, UK; 2 Sunderland Eye Infirmary, Queen Alexandra Road, Sunderland SR2 9HP, UK

## Abstract

**Background:**

Relatively little is known about the incidence of prescribing errors and there has been no work on this in a single specialty ophthalmic hospital. Knowing where and when errors are most likely to occur is generally felt to be the first step in trying to prevent these errors. This study is an attempt in, the setting of an eye hospital, to try to identify and attribute these medication errors.

**Methods:**

The study setting was a single specialty eye hospital geographically separated from the main general hospital. Pharmacists prospectively recorded the number of errors of prescribing during a 4 week period at an eye hospital in UK. The errors were categorised as error of prescription writing or drug error. Potential significance of the errors was not addressed.

**Results:**

Overall 144/1952 (8%)prescription sheets had errors. 7% of the total errors were errors of prescription writing while 1% were drug errors. The majority of errors were made by junior doctors and no drug errors were made by senior doctors. The outpatients department had by far the highest prevalence of errors.

**Conclusion:**

Certain areas within the hospital and certain grades of staff are more prone to drug errors. Further study is required to look at the reasons why this is so and what systems can be put in place to reduce these errors.

## Background

Medication errors are some of the commonest types of medical errors.[1] Although the true incidence of medication errors for UK is not known, a pilot study identified errors of prescribing (rather than of dispensing) as the commonest cause of medication errors in the study hospital.[2]

The Department of Health has recommended that by 2005 serious errors in prescribed drugs should be reduced by 40%.[3] Since errors of prescribing are the commonest form of avoidable medication errors, it is the most important target for improvement.[4,5]

One drug or dosage, which may be harmless in one patient, may cause serious harm to another. Consequently, it is important that all branches of medicine try to identify and study their own sources of prescribing errors. To the best of our knowledge, a study identifying medication errors arising in an eye hospital has not been previously published.

The aim of this study was to estimate the number of medication errors, where they most commonly occur and the doctors most likely to commit them.

## Methods

The study was conducted at Sunderland Eye Infirmary. This is a single specialty hospital situated two miles from the main general hospital. It contains its own self-contained medical records, theatres, accident and emergency department (which is walk-in), in-patient ward and pharmacy. The outpatients department has clinics that cover all the main specialties within ophthalmology.

For the purpose of the study, the hospital was divided into the following areas

• The out patients department (OPD)

• Accident and Emergency (A&E)

• Day case centres (including day-case theatres and laser rooms)

• In patient ward

From this latter group only discharge summaries were assessed for errors. In patient drug charts were not assessed

The study was conducted prospectively over a four week period starting on 1^st ^July 2002. It was performed during the working hours of the pharmacy (9 am to 5 pm) and therefore evenings and weekends were excluded.

In order to replicate normal working conditions as much as possible, prescribing doctors were not informed of the study. The participating pharmacists were introduced to the study protocol over two half-day sessions. Errors on the prescription were identified by three dispensing pharmacists (all prescriptions at the time of the study were hand written).

The pharmacists recorded on sheets the errors as either;

1) errors of prescription writing or

2) drug related errors.

Prescription writing errors consisted of :-

• Incorrect patient details e.g. patient name, hospital number or date of birth

• Illegibility

• Incorrect format

• Scripts where the prescribing doctor could not be identified.

Once a prescription writing error was identified, the script was not further assessed for drug related errors. If there was no prescription writing error identified then the script was assessed for drug related errors and these were defined as:-

• Incorrect drug dose or timing

• Incorrect route of administration.

Since the dispensing pharmacists did not have access to patient notes from A&E, OPD and day case centres, they were unable to comment on the correctness of prescribing decision in each situation.

For the purpose of this study, senior doctors included consultants, staff grade and associate specialists. Junior doctors were senior house officers, specialist registrars and research fellows.

## Results

Over the study period, a total of 1952 prescription orders were received by the pharmacy with a total of 3402 medication orders – although the majority of prescriptions only had a single medication order. The contribution to the total prescriptions from the different parts of the hospital and the breakdown of errors is shown in Table 1.

Overall 159 (8%) of the 1952 prescriptions had at least one error of writing or a drug error. The breakdown of these errors was as follows:-

### Errors of prescription writing

144/1952 of the scripts had incorrect formats or were illegible. They constituted 7% of the total errors in this study.

In 18/144 (13%) of them the prescribing doctor could not be identified. Of the remaining 126 prescriptions, senior doctors made 46% of the errors compared to 54% made by the junior doctors.

The inpatient discharge scripts did not have any errors of prescription writing. A&E, OPD and day case contributed to 56(39%), 64(45%) and 24(17%) of the errors respectively.

### Drug related errors

were identified in 15/1808 (this being the total number of prescriptions **without **written errors). This represented 1% of the prescription orders. 8/15 of the errors (53%) were of incorrect dosage while the remainder were either incorrect route of administration or incorrect frequency. All the errors, in this category, were made by junior doctors at the hospital. The distribution of writing and drug errors between senior and junior doctors is shown in Table 2.

**Figure 1:**
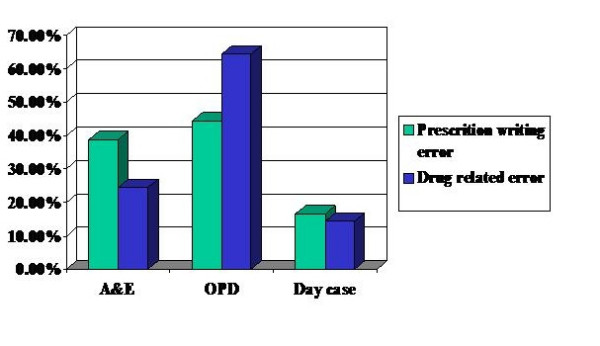
The contribution to prescribing errors from different areas of the hospital

Distribution of the prescription writing errors and drug related errors with respect to part of the hospital in which they occurred are illustrated in Figure 1. It can be seen that no errors were identified from the in-patient discharge summaries while 25%, 64% and 14% were the percentage distribution of these prescriptions from A&E, OPD and day case areas respectively.

As discharge summaries (which include discharge ophthalmic medications only and not the patients' non-ophthalmic drugs) were found not to have any errors of prescription writing or drug related errors, it was felt necessary to double-check this finding. Discharge summaries were then further analysed by one of the investigators (KM) and they were also cross-checked with the in-patient notes. There were found to be a total of 130 discharge summaries during the study period and it was confirmed that there were no prescription writing or drug related prescribing errors in theses summaries. It as also noted that 103 (79%), of the 130 discharge summaries, were written by senior doctors.

## Discussion

The prescribing of medicines is an integral part of the provision of health and represents a relatively safe, effective and inexpensive mode of treatment.[6] However a study in the US has identified medication errors as the cause of harm in 1–2% of all hospital admissions.[7]

**Figure 2:**
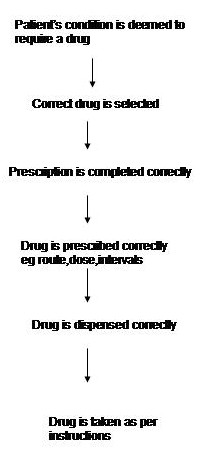
The steps involved in a patient receiving the correct medication

The end point of a patient receiving a medication involves a series of steps (illustrated in figure 2) and errors can be made at any point in this process.

This study addresses only two links in the chain i.e. correct completion of the prescription and drug errors. We did not assess treatment decisions or dispensing errors. However, if prescribing errors are to be reduced (the Department of Health target is by 40% by 2005) it is important to know where they are occurring, who is committing them and in what situations. In effect it is necessary for each specialty, hospital or team to know where their 'weak points' are in order that they can put in the appropriate defences. This study is an attempt to identify some of the weak points in a single specialty ophthalmic hospital.

The overall rate of errors in our study was 8%, but as only one error was counted per script, the actual true number of errors may have been greater. The literature suggests that between 1 and 38% of all medications are administered in error.[8] Dean et al,[1] found a prescribing error rate of 1.5% in one UK general hospital. This wide variation is partly due to differences in definition of drug errors and partly due to problems of reporting (Osborne at al suggest that 25% of medication errors are not reported).[9] A standardised definition such as that suggested by Dean and Barber[10] is essential if different units, specialties and countries are to be compared. Similarly a rigorous and reasonably standardised collecting system is necessary – although no system will be foolproof, it would at least achieve a level of consistency.

Ophthalmology represents a specialty with a wide range of differing medications and different routes of administration. Most drugs are given as drops or ointment into the conjunctival sac but many are given orally and intravenously. Medications range from the very safe e.g. artificial tears to the potentially serious e.g. intravenous steroids and oral cytotoxics. Ophthalmic medications also have the potential, unlike in most other specialties, to cause sight threatening side effects e.g. topical steroids. Even topical medications can have serious side-effects because of absorption from the highly vascular nasolacrimal system and the lack of first-pass liver metabolism. A good example of this is topical β-blockers used to lower the intraocular pressure in glaucoma but which can exacerbate or even cause bronchospasm.[11] All medication errors are, of course, potentially serious in that they may indicate flaws in a system that may allow more serious errors to occur.

Our study shows that the clinical area with the highest incidence of prescribing error rates was the out-patients department. This finding is somewhat surprising both in its scale (50% of all scripts) and its location. We had felt, prior to commencing the study, that the accident and emergency department would be the most likely area to produce prescribing errors as it has a high throughput, a rapid turnover of patients and is generally staffed by the more junior doctors in the department.

The actual prescribing error rate in A&E was only 5% and we now suspect this relatively low rate of error was for two reasons:-

1. There is only a narrow range of drugs that are usually used in the ophthalmic A&E setting

2. Because of the set-up of the A&E department, many patients give their prescriptions to an A&E nurse before going to pharmacy and it may be that some errors are being corrected at this stage.

Interestingly, the day case prescription sheets are also for a small range of medications and many are checked by the nurses prior to the patient being sent to pharmacy. This gives the same proportion of errors (5%) as in A&E.

The in-patient ward discharge prescriptions had no errors in this study. There may be two reasons for this:

1. All the ward discharge prescriptions are checked by the nursing staff prior to going to the pharmacy

2. All the ward discharge were written by the senior medical staff

The out-patient setting seems to differ from the other areas in two main respects:-

1. there is a much wider range of drugs being prescribed – both topical and systemic

2. it would be unusual for a nurse to handle the prescription prior to the patient presenting it to the pharmacy.

We feel that it is these differences that, at least partly, explain the differences between the rates of error between OPD and the other areas. However a 50% error rate seems very high and we are currently addressing other possible reasons for this.

The figures indicate that errors of prescription writing were much more common than drug errors. This is as we would expect – not only from the fact that the study did not count drug errors if writing errors had occurred. There are a number of ways to write a prescription wrongly but only one or two ways to commit a drug error. There also seems, from our results, to be a tendency to get drug doses/timings correct but to cut as many corners as possible to speed up writing prescriptions. This is also shown by the fact that both junior and senior doctors made far more writing errors than drug errors. Seniors made no drug errors -perhaps reflecting their greater experience of using the drugs but made just as many writing errors as the juniors. Prescription writing errors are a rich source of medication errors and one that could be almost completely removed with electronic prescribing.[12,13]

This study is an attempt to represent a 'snap-shot' of medication errors at a single institution over a period of four weeks. It is therefore important to be cautious about the generalisability of the results – the study hospital may have unique features or the study time period may have been an exceptional one. The literature indicates a wide variation in estimates of medication errors and further work is needed to compare different specialties, hospitals and countries for a true picture to emerge. We have not addressed the potential clinical significance of the errors made in this study but again this needs further study including agreement over definitions of serious and less serious errors with ophthalmic medications. It is possible that our study has underestimated the total number of drug errors by not looking for any further errors in the scripts that had transcription errors. These prescriptions with writing errors were probably filled in haste and may therefore, have been more likely to contain drug errors resulting in an underestimate of this category.

In conclusion, this study found an overall medication error rate of 8%. Most of these errors occurred during the writing of the prescriptions. Junior doctors were more likely to make errors than were senior doctors. The out-patients department had a very high rate of medication errors and this is a finding that needs further study. It is important to look into the reasons why these discrepancies exist and to address them in a non-punative way and to look for ways in which the prescribing systems can be changed to take account of these weak points.

Electronic prescribing is soon to be used in the study hospital and it is likely that this will significantly reduce the number of prescription writing errors and drug errors.[14] We will repeat this study, using a similar format, after the introduction of electronic prescribing to see if this has occurred.

## Conclusion

Some areas of the hospital and certain grades of doctors are more prone to medication errors compared to others. These specific areas need to be targeted when planning and implementing any changes to improve services.

## Competing interests

The author(s) declare that they have no competing interests.

## Authors' contributions

SGF conceived the study, and participated in its design and co-ordination. KM carried out the data collection and statistical analysis. All authors have read and approved the final manuscript.

## Pre-publication history

The pre-publication history for this paper can be accessed here:



## Figures and Tables

**Table 1:** Total prescriptions in study period and the numbers with errors identified by study pharmacists

	TOTAL NO PRESCRIPTIONS IN AREA OVER THE STUDY PERIOD	NUMBER WITH ERRORS OF PRESCRIPTION WRITING	NUMBER WITH DRUG ERRORS	TOTAL NUMBER OF SCRIPTS WITH ERRORS	% OF SCRIPTS WITH ERRORS
OPD	**144**	**64**	**9**	**73**	**50%**
A&E	**1117**	**56**	**4**	**60**	**5%**
DAY	**561**	**24**	**2**	**26**	**5%**
CASE					
WARD	**130**	**0**	**0**	**0**	**0%**
	**1952**	**144 (7%)**	**15 (1%)**	**159**	**8%**

**Table 2:** Distribution of errors between senior and junior doctors.

**GRADE OF DOCTOR**	**ERRORS OF PRESCRIPTION WRITING**	**DRUG RELATED ERRORS**
**SENIOR**	58 (46%)	0%
**JUNIOR**	68 (54%)	15 (100%)
